# Spontaneous remission of adult-onset minimal change nephrotic syndrome associated with influenza B infection: a case report

**DOI:** 10.1186/s12882-018-0961-y

**Published:** 2018-07-04

**Authors:** Ayumi Haruki, Eiji Ishikawa, Kan Katayama, Takayasu Ito, Takuya Hiramoto, Mika Fujimoto, Tomohiro Murata, Masaaki Ito

**Affiliations:** Department of Cardiology and Nephrology, Mie University Graduate School of Medicine, 2-174 Edobashi, Tsu, Mie 514-8507 Japan

**Keywords:** Minimal change nephrotic syndrome, Spontaneous remission, Type B influenza infection

## Abstract

**Background:**

While the majority of adult-onset minimal change nephrotic syndrome (MCNS) is a primary or an idiopathic form of disease, it can also occur as a secondary form. Reports on the spontaneous remission of MCNS are rare since the condition is typically treated with corticosteroids. We herein describe the spontaneous remission of adult-onset MCNS in a patient who developed nephrotic syndrome after type B influenza infection.

**Case presentation:**

A 50-year-old woman experienced fever, cough, malaise, and low back pain, which had persisted for 6 days before she presented to our hospital, and edema of the face and limbs, which had persisted for 5 days before her presentation. She was diagnosed with type B influenza infection and later exhibited an exacerbation of facial edema, decreased urine output, and a high level of proteinuria. She was referred to our department after the diagnosis of nephrotic syndrome. On admission, her proteinuria level was 20.88 g/gCr and her selectivity index value was 0.13. The examination of a kidney biopsy specimen obtained on the fourth day of hospitalization under a light microscope revealed minor abnormalities. An immunofluorescence showed only nonspecific granular IgM deposits in the mesangium. Electron microscopy showed extensive foot process effacement without any immune complex deposits. Based on these findings, the patient was diagnosed with MCNS. After admission, the proteinuria decreased to 0.06 g/gCr with rest and sodium restriction (6 g/day) alone; a complete remission from nephrotic syndrome was observed at approximately 2 weeks after the onset of symptoms. There have been no signs of recurrence of nephrotic syndrome in the one years since.

**Conclusion:**

We experienced a rare case in which spontaneous remission of MCNS occurred within a short period of 2 weeks after influenza B infection. When patients present with nephrotic syndrome after an infection, it is necessary to consider MCNS in the differential diagnosis, which also includes post-infectious glomerulonephritis and the acute exacerbation of IgA nephropathy.

## Background

Minimal change nephrotic syndrome (MCNS) is known to account for 10–15% of nephrotic syndrome in adults [1]. While the majority of adult-onset MCNS is a primary or idiopathic form of disease, it can also occur as a secondary form due to infection, medication, tumor, or other kidney diseases [2]. Although spontaneous remission is known to occur in MCNS, there are few recent reports of this phenomenon because initial treatment with corticosteroids has been widely-accepted. We herein report a case of spontaneous remission of MCNS in a patient who developed nephrotic syndrome after contracting type B influenza.

## Case presentation

The patient was a 50-year-old woman with a chief complaint of edema. She had previously been healthy, and previous physical examinations revealed no abnormal urinalysis results. She experienced fever, cough, malaise, and low back pain for 6 days and edema of the face and limbs for 5 days before visiting our hospital. She was diagnosed with type B influenza by a local physician 3 days prior to visiting our hospital. The patient later exhibited an exacerbation of facial edema, decreased urine output, and a high level of proteinuria. She was subsequently diagnosed with nephrotic syndrome and was referred to our department.

The physical findings on admission were as follows: height, 150.1 cm; weight, 48.3 kg; body mass index, 21.4; body temperature, 36.2 °C; blood pressure, 109/83 mmHg; and oxygen saturation on room-air, 99%. In addition, the patient experienced facial edema and pitting edema of both lower legs. The laboratory findings on admission were as follows: total protein, 5.2 g/dL; albumin, 2.0 g/dL; blood urea nitrogen, 13 mg/dL; creatinine, 0.84 mg/dL; estimated glomerular filtration rate, 56.5 mL/min/1.73 m^2^; total cholesterol, 308 mg/dL; immunoglobulin G (IgG), 1320 mg/dL; IgA, 354 mg/dL; IgM, 192 mg/dL; IgE, 2966 IU/mL; complement 3, 83.3 mg/dL (normal range 65–141 mg/dL); complement 4, 40.9 mg/dL (normal range 13–40 mg/dL); and total hemolytic complement (CH50), 40.5 U/mL (normal range 31–48 U/mL). The patient was negative for hepatitis B virus surface antigen, hepatitis C virus antibodies, and human immunodeficiency virus antibodies. She had previously been infected with parvovirus B19. Her proteinuria level was 20.88 g/gCr, her selectivity index value was 0.13, and her urine sodium level was 8 mEq/L. Urine sediments showed 1–4 red blood cells per high-power field without any abnormal casts. There were no notable abnormalities electrocardiography or chest X-ray images.

**Fig. 1:**
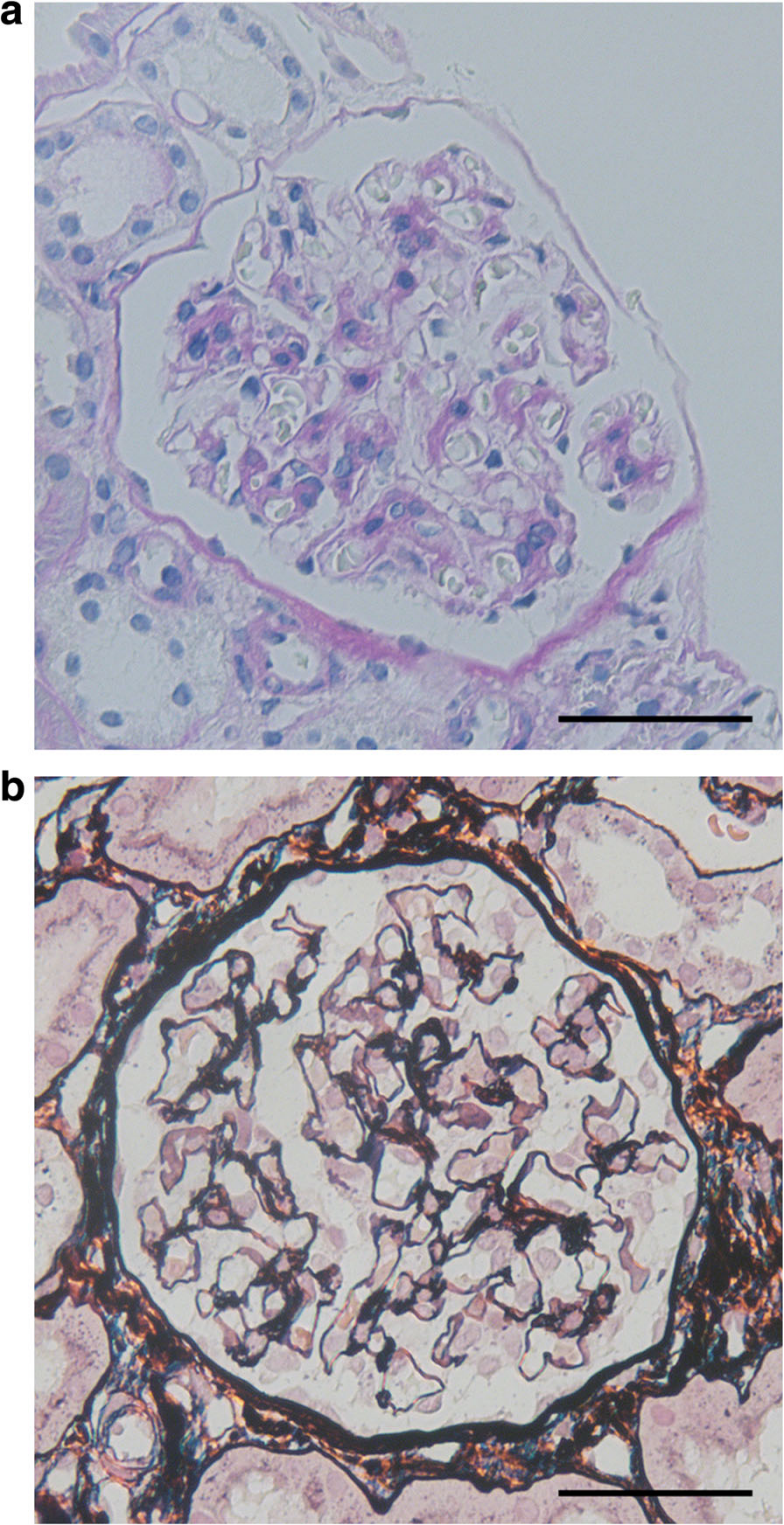
The light microscopic examination of a kidney biopsy specimen. **a** Periodic acid-Schiff staining did not show mesangial cell proliferation or increases in mesangial matrix. **b** Periodic acid methenamine silver staining did not show thickening, double-contouring, or spike formation of the basement membrane. Scale bars, 50 μm

**Fig. 2:**
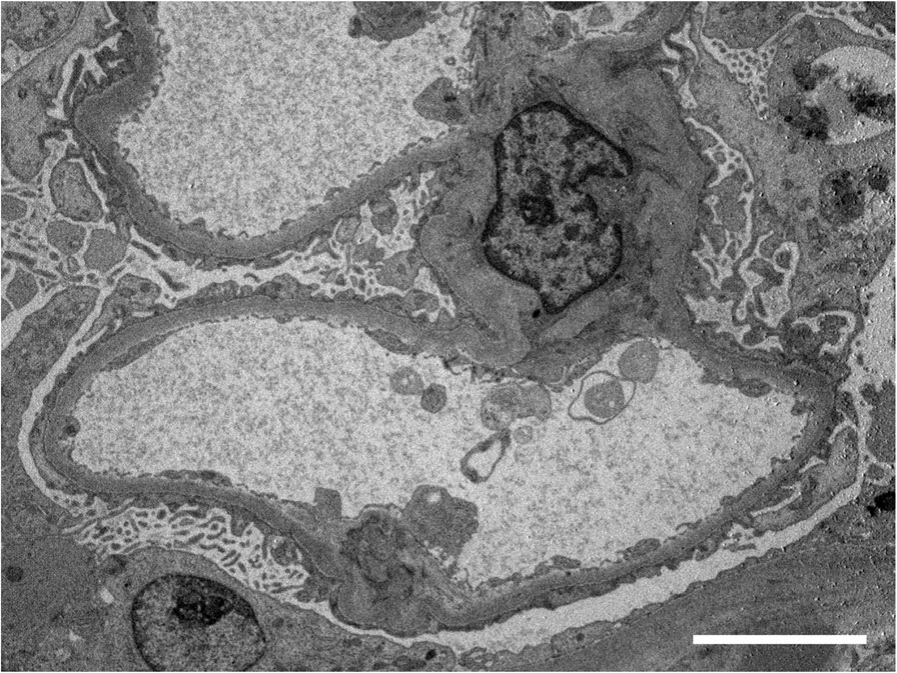
The electron microscopic findings. No immune complex deposits were observed. Extensive foot process effacement was observed. Scale bar, 5 μm

A kidney biopsy performed on the fourth day of hospitalization under suspicion of MCNS, IgA nephropathy, or post-streptococcal acute glomerulonephritis did not show glomerular basement membrane thickening, or endocapillary or mesangial cell proliferation (Figs. 1 a and b). An immunofluorescence study showed nonspecific granular IgM deposits in the mesangium. Electron microscopy showed extensive foot process effacement, but immune complex deposits were not observed (Fig. 2). Based on the above findings, the patient was diagnosed with MCNS.

**Fig. 3:**
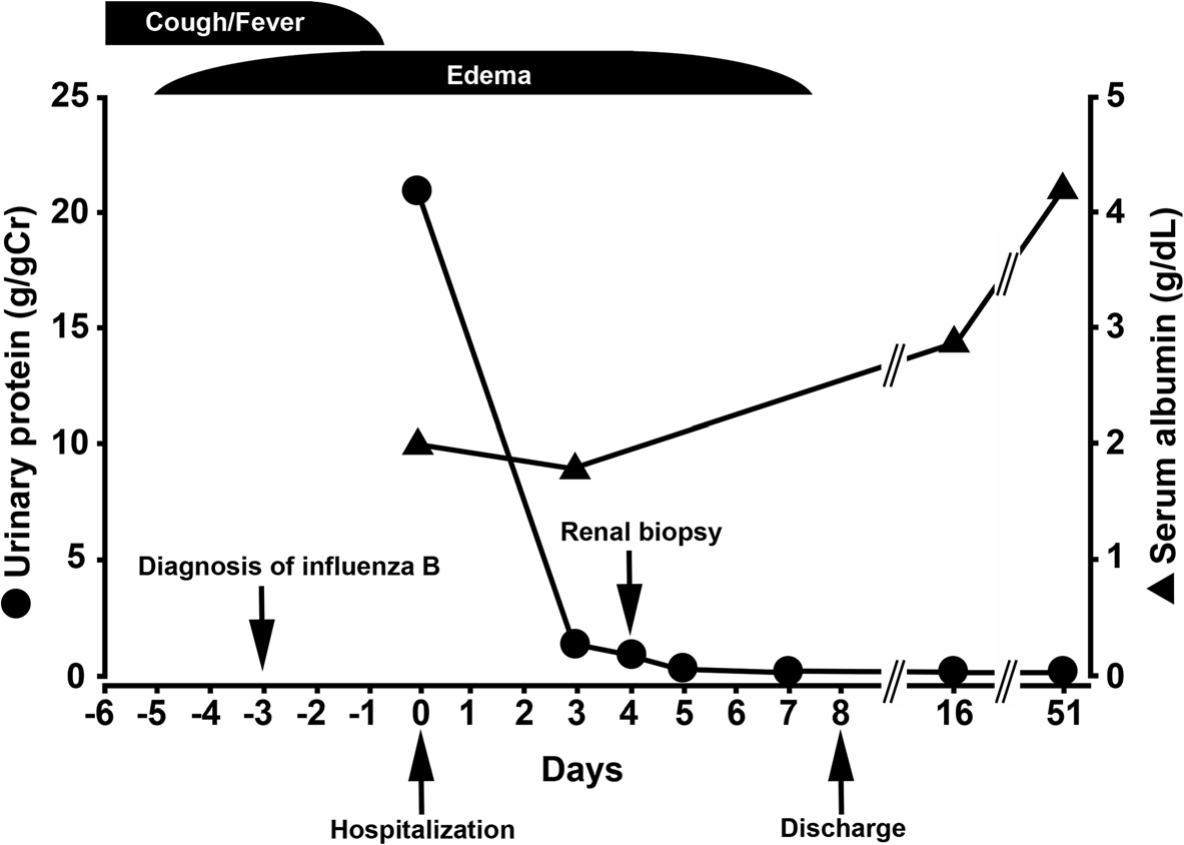
The clinical course. Spontaneous remission of acute-onset generalized edema and high levels of proteinuria that occurred after influenza infection. Spontaneous remission was observed after approximately 2 weeks

After admission, the patient’s proteinuria decreased to 0.06 g/gCr with rest and sodium restriction (6 g/day) alone, and a complete remission from nephrotic syndrome was observed at approximately 2 weeks after the onset of symptoms. No recurrence of nephrotic syndrome was observed at an outpatient visit one month later (Fig. 3). No recurrence has been noted in the one years since.

## Discussion

In the present case, the patient developed nephrotic syndrome following an influenza B infection and was diagnosed with MCNS based on her clinical course and the results of the histopathological examination of a kidney biopsy specimen. With careful observation in our hospital, spontaneous remission occurred within two weeks after the onset of symptoms without corticosteroid treatment.

Since the etiology of nephrotic syndrome in adults is more diverse than in children, the indication for a renal biopsy differs between adults and children [3]. Typically, nephrotic syndrome in children achieve complete remission within weeks, whereas that in adults require months. Therefore, a renal biopsy is not routinely performed in children. In the present case, the patient’s proteinuria was 1.59 g/gCr, and her serum albumin level was 1.8 g/dL on the day before the renal biopsy. Although her urinary protein was improving, it was still over 1 g/gCr. It was necessary to make a differential diagnosis between MCNS and IgA nephropathy or post-streptococcal acute glomerulonephritis, which carried a risk of worsening immediately after infections. Accurately diagnosing the etiology of nephrotic syndrome strongly influences the subsequent treatment options.

The patient was diagnosed with influenza by her previous physician using a quick diagnostic kit (Quick-Navi™-Flu, Otsuka Pharmaceutical Co., Ltd., Tokyo, Japan). A serological diagnosis of the influenza antibodies was not conducted. However, this quick diagnostic kit has a specificity of 98.2% and a sensitivity of 62.3% [4], and the likelihood of a false-positive result is low when the test result is positive. Thus, together with the symptoms that the patient exhibited, it is highly likely that she was affected by influenza.

We lacked data on the proteinuria on the day of the influenza B infection diagnosis because no urinalysis was performed at the local clinic where her influenza B infection was diagnosed. However, she had undergone an annual health checkup every year before the occurrence of nephrotic syndrome and had shown no proteinuria four months before the influenza B infection. She was therefore deemed to have been completely healthy shortly before developing a high fever as her first symptom of influenza B infection.

Although reports on the association between influenza infection and MCNS are extremely limited, the association between the viral infection of upper respiratory tract including influenza virus and the recurrence of childhood nephrotic syndrome has previously been reported [5]. Another article reported that MCNS recurred after a patient contracted influenza [6]. Those authors suggested the possibility of an unknown rare genetic or environmental predisposition that was essential for the pandemic influenza virus to cause podocyte damage and nephrotic syndrome [6]. A case in which MCNS developed after an influenza vaccination has also been reported [7]. Furthermore, nephrotic syndrome was reported to develop in a pediatric patient after the patient contracted influenza [8].

Among the MCNS cases in which spontaneous remission was reported, there were cases of secondary MCNS that developed after viral infections such as rubeola or varicella [9], after receiving medication [10], and after an influenza vaccination [7]. In addition, the spontaneous remission of MCNS has been reported in cases in which complications of viral infections such as measles [11], varicella [12], and hepatitis A virus [13] occurred while a patient was affected with MCNS. Although the pathogenesis of MCNS is considered to be multifactorial, attributed to both intrinsic and extrinsic factors [14], one article has suggested that T-cell dysfunction and immunologic abnormalities of permeability factors and CD80 are involved [15]. A previous report showed that the serum IgE level was high in MCNS patients compared to normal controls at the diagnosis and in remission and suggested some type of immune abnormality in MCNS patients [16]. In the present case, the serum IgE level was high at 2966 IU/mL and remain high even after remission (527 IU/mL). We hypothesized that the present case had already been predisposed to MCNS and changes in immunity due to the influenza B infection, leading to the onset of MCNS, and remission was thought to have occurred as a result of the immediate normalization of the immune system after recovery from influenza B infection.

The involvement of medications, including nonsteroidal anti-inflammatory drugs, lithium, interferon, ampicillin, rifampicin, D-penicillamine, and pamidronate, is known to cause secondary MCNS [2]. In the present case, although the patient did not take routine oral medications, other medications, including garenoxacin, Maobushisaishinto (a Chinese medicine), and acetaminophen were prescribed after she was diagnosed with influenza. Although these medications may have led to the onset of MCNS, we believe that the likelihood of that was low since the patient had exhibited edema prior to taking the prescribed medications.

The spontaneous remission of MCNS was not uncommon in the 1940s to 1950s before corticosteroids were used as the standard treatment [17]. As corticosteroids became the standard treatment for MCNS, the actual situation regarding spontaneous remission became unclear since corticosteroids are administered at a relatively early time point. Consequently, reports on the spontaneous remission of MCNS are rare. Nolasco et al. reported spontaneous remission in 6 of 89 patients with adult-onset MCNS (6.7%) [18], and Shinzawa et al. similarly reported spontaneous remission in 9 of 142 patients (6.3%) [19]. Spontaneous remission is also known to occur in cases in which MCNS frequently recurs: there are reports of spontaneous remission in both initial and recurrent adult-onset MCNS. Spontaneous remission occurred in 6% of 50 patients with initial MCNS, and in 33% of 51 patients with recurrent MCNS [20, 21]. In previous reports on the spontaneous remission of MCNS, no specific trends were observed with regard to the duration until remission, age, sex, cause, and clinical findings [18–21].

## Conclusion

Reports on initial MCNS after contracting influenza are rare. We experienced a case in which spontaneous remission occurred in a short period of 2 weeks. In cases of nephrotic syndrome after infection, it is necessary to consider MCNS as well as post-infectious glomerulonephritis and an acute exacerbation of IgA nephropathy in the differential diagnosis. Moreover, spontaneous remission may occur in such cases; thus, decisions concerning the administration of corticosteroids should be made carefully. The further accumulation of cases is necessary for future study.

## Abbreviations

CH50: total hemolytic complement
IgG: immunoglobulin G
MCNS: minimal change nephrotic syndrome
